# Seizures in a cat with metabotropic glutamate receptor 1 (mGluR1) antibodies: a case report

**DOI:** 10.3389/fvets.2026.1894456

**Published:** 2026-08-12

**Authors:** Magdalena Putzer, Alina Hörmann, Viktoria Stegfellner, Holger A. Volk, Jasmin N. Neßler

**Affiliations:** Department of Small Animal Internal Medicine and Surgery, University of Veterinary Medicine, Hannover, Germany

**Keywords:** autoantibody, autoimmune encephalitides, behavioral change, feline epilepsy, mGluR1

## Abstract

Autoimmune encephalitis associated with neuronal autoantibodies is increasingly recognized in veterinary medicine. In cats, previously reported antibodies have primarily targeted components of the voltage-gated potassium channel complex, such as leucine-rich glioma-inactivated 1 (LGI1) and contactin-associated protein-like 2 (CASPR2). Anti-metabotropic glutamate receptor 1 (mGluR1) antibodies are well described in human medicine but have not previously been reported in cats. A 5.5-month-old female domestic shorthair cat was presented for recurrent epileptic seizures and episodic behavioral abnormalities, including pica. Neurological examination revealed mild pelvic limb ataxia. Based on the history of epileptic seizures and behavioral abnormalities, the neuroanatomical localization was consistent with the forebrain. Magnetic Resonance Imaging (MRI) of the brain and cerebrospinal fluid (CSF) analysis were unremarkable. Infectious disease testing from CSF, including polymerase chain reaction (PCR) assays for Feline Coronavirus, *Toxoplasma gondii*, Feline Herpesvirus, and Rustrela virus, was negative. Neural antibody testing using tissue-based and cell-based assays identified an elevated serum mGluR1 antibody titer (1:80; reference <1:20), whereas cerebrospinal fluid and tissue-based assays were negative. Testing for other neuronal autoantibodies, including LGI1 and CASPR2, was negative. Following optimization of antiseizure therapy, the cat showed clinical improvement, accompanied by a decrease in the serum mGluR1 antibody titer. This report describes the first detection of mGluR1 antibodies in a cat with neurological disease and suggests a potential association between mGluR1 autoimmunity and epileptic seizures and episodic behavioral abnormalities in a cat.

## Introduction

Epilepsy is a neurological disorder characterized by recurrent epileptic seizures resulting from sudden, excessive, and synchronous neuronal network activity, which may manifest with motor, autonomic, and/or behavioral signs (1). Epilepsy can be classified as primary (idiopathic) or secondary, with the latter encompassing structural brain disease (2). According to the International Veterinary Epilepsy Task Force guidelines for dogs, which are widely applied to cats in the absence of species-specific recommendations, identification of the underlying etiology requires a stepwise diagnostic work-up consisting of physical and neurological examination, laboratory testing including hematology, serum biochemistry and bile acids, urinalysis, Magnetic Resonance Imaging (MRI) of the brain and cerebrospinal fluid (CSF) analysis (3). Within the category of structural epilepsy, the most common causes are inflammatory disorders (44%), followed by neoplasia (38%) and vascular etiologies (18%) (4).

Autoimmune encephalitis comprises a group of non-infectious inflammatory brain disorders that may present with epileptic seizures but can also manifest with a wide range of neurological deficits and behavioral abnormalities. These conditions are often, though not invariably, associated with neuronal autoantibodies (5, 6).

Several autoimmune encephalitides associated with neuronal or glial autoantibodies have been described in the veterinary literature, particularly in dogs. These include antibodies against the GABA_A_ receptor (7), N-methyl-D-aspartate receptor 1 (NMDAR1) (8), glial fibrillary acidic protein (GFAP) (9, 10), myelin basic protein (MBP) (11), and tripartite motif–containing protein 46 (TRIM46) (12), resulting in a variety of neurological deficits. Autoantibody-associated encephalitis has also been described in cats, most commonly in association with antibodies directed against voltage-gated potassium channel (VGKC) complexes, targeting leucine-rich glioma-inactivated 1 (LGI1) and contactin-associated protein-like 2 (CASPR2) (13). Furthermore, a single case report described antibodies against the netrin-1 receptor deleted in colorectal cancer (DCC) in a cat with limbic encephalitis and hippocampal necrosis (14).

Metabotropic glutamate receptors play an important role in the modulation of synaptic transmission throughout the central nervous system. A variety of receptor subtypes influence neuronal excitability, synaptic plasticity, neuroinflammatory processes, and glial function (15). The metabotropic glutamate receptor 1 is highly expressed in Purkinje cells of the cerebellar cortex but is also present in other brain regions, including the hippocampus, thalamus, basal nuclei, and midbrain structures (16). The mGluR1 is a G-protein—coupled receptor that activates intracellular signaling pathways involving phospholipase C, leading to intracellular calcium release and activation of protein kinase C. Through these mechanisms, mGluR1 modulates neuronal excitability and contributes to synaptic plasticity (15).

Studies in mGluR1 knockout mice demonstrate the critical role of this receptor in synaptic plasticity and cerebellar function. These animals show abnormalities in hippocampal long-term potentiation and deficits in cerebellar long-term depression (a type of synaptic plasticity considered critical for motor learning), accompanied by impairments in learning, motor coordination, and associative learning (17, 18). The functional importance of mGluR1 is further reflected by the recognition of mGluR1-associated autoimmune encephalitis in human medicine. It most commonly presents with subacute cerebellar ataxia, likely reflecting impaired Purkinje cell function, although additional neurological manifestations have been described, including dysarthria, epileptic seizures, behavioral changes and movement disorders (19–21).

While mGluR1 antibodies have been identified in dogs with idiopathic generalized tremor syndrome (10) their presence has not yet been reported in cats with neurological disease. Although generalized tremors were the pre-dominant clinical sign in the reported canine cohort, one affected dog also presented with generalized tonic–clonic epileptic seizures, indicating that the clinical spectrum of mGluR1-associated disease may extend beyond a purely cerebellar phenotype (10). To the authors' knowledge, this case therefore represents the first report of mGluR1 antibodies in a cat with neurologicaldisease.

## Case description

A 5.5-month-old, female, domestic shorthair cat was found as a stray when she was a few days old and was presented with a history of cluster seizures. According to the caretaker, the first epileptic seizure had been observed at approximately 6 weeks of age. Episodic behavioral abnormalities were reported, including excessive suckling, ingestion of cat litter, and chewing or eating inedible objects consistent with pica. The described seizure semiology consisted of sudden arousal from rest, staring episodes, aggressive behavior, followed by generalized tonic–clonic epileptic seizures with autonomic signs.

Prior to referral, the cat had been treated with phenobarbital for approximately 2 months at a dosage of 6.6 mg/kg per os (PO) twice daily without significant clinical improvement. Despite the high dosage, the peak serum phenobarbital concentration was subtherapeutic at 9.1 mg/L (desired therapeutic concentration: 15–35 mg/L).

General physical examination was unremarkable. Neurological examination revealed mild proprioceptive pelvic limb ataxia without any other neurological abnormalities. Based on the history of epileptic seizures and behavioral abnormalities, neuroanatomical localization was consistent with a forebrain lesion. The mild ataxia was considered most likely to represent an side effect of phenobarbital therapy. Hematology and serum biochemistry demonstrated mild leukocytosis (12.06 × 10^3^/μL; reference range 6–11 × 10^3^/μL), mild thrombocytosis (557 × 10^3^/μL; reference range 200–550 × 10^3^/μL), mild elevation in creatine kinase activity (133 U/L; reference range 0–130 U/L), mildly increased total protein (8.56 g/dL; reference range 6–8 g/dL), and borderline hyperkalemia (4.83 mmol/L; reference range 3–4.8 mmol/L). Fasted blood ammonia and bile acid concentrations were within the normal range. Postprandial bile acid concentration was not measured because the cat required sedation for the subsequent diagnostic work-up and was therefore not fed. Serologic testing for feline immunodeficiency virus and feline leukemia virus was negative.

Magnetic Resonance Imaging of the brain was performed using a 3-Tesla magnet (Achieva, Philips Medical Systems, Best, The Netherlands). The imaging protocol included T2-weighted and pre- and post-contrast T1-weighted sequences acquired in transverse, sagittal, and dorsal planes, as well as transverse T2-weighted fluid-attenuated inversion recovery (FLAIR), diffusion-weighted imaging (DWI), and apparent diffusion coefficient (ADC) maps. No intracranial abnormalities were identified. Cerebrospinal fluid was obtained from the cerebellomedullary cistern. The CSF analysis revealed a total nucleated cell count of 0 cells/μL (reference range 0–5 cells/μL), an erythrocyte count of 1 cell/3 μL (reference range 0 cells/3 μL), and a protein concentration of 10 mg/dL (reference range 0–25 mg/dL). Infectious disease testing from CSF, including polymerase chain reaction (PCR) assays for Feline Coronavirus, Toxoplasma gondii, Feline Herpesvirus, and Rustrela virus, was negative.

Because routine diagnostic investigations, including hematology, serum biochemistry, MRI, CSF analysis, and infectious disease testing, failed to identify an underlying cause for the recurrent epileptic seizures and behavioral abnormalities neural antibody testing was pursued to investigate a possible autoimmune etiology. Serum and CSF samples were collected, stored at −20 °C immediately after collection, and transported frozen on dry ice to MVZ Labor Krone GmbH (Bad Salzuflen, Germany) for neural antibody testing. Analysis included a commercially available human autoimmune encephalitis antibody panel using cell-based indirect immunofluorescence assays (human embryonic kidney cells; Euroimmun, Lübeck, Germany) directed against glutamic acid decarboxylase 65 (GAD65), NMDAR, GABAAR, γ-aminobutyric acid B receptor (GABABR), immunoglobulin-like cell adhesion molecule 5 (IgLON5), α-amino-3-hydroxy-5-methyl-4-isoxazolepropionic acid receptor 1/2 (AMPAR1/2), dipeptidyl-peptidase-like protein 6 (DPPX), LGI1, CASPR2, glycine receptor (GlyR), metabotropic glutamate receptor 5 (mGluR5), and mGluR1, together with a mouse brain tissue-based indirect immunofluorescence assay (Table 1). Human samples with different antibody reactivities served as positive controls. The only abnormal finding was an elevated serum mGluR1 antibody titer of 1:80 (reference < 1:20) detected in the cell-based assay, while CSF was negative.

**Table 1:** Results of neural antibody testing using a commercially available human autoimmune encephalitis cell-based assay.

Tested antibody	Serum titer	Reference in serum	CSF titer	Reference in CSF
GAD65	< 1:20	< 1:20	< 1:1	< 1:1
NMDAR	< 1:20	< 1:20	< 1:1	< 1:1
GABAAR	< 1:20	< 1:20	< 1:1	< 1:1
GABABR	< 1:20	< 1:20	< 1:1	< 1:1
IgLON5	< 1:20	< 1:20	< 1:1	< 1:1
AMPAR1/2	< 1:20	< 1:20	< 1:1	< 1:1
DPPX	< 1:20	< 1:20	< 1:1	< 1:1
LGI1	< 1:20	< 1:20	< 1:1	< 1:1
CASPR2	< 1:20	< 1:20	< 1:1	< 1:1
GlyR	< 1:20	< 1:20	< 1:1	< 1:1
mGluR5	< 1:20	< 1:20	< 1:1	< 1:1
mGluR1	1:80	< 1:20	< 1:1	< 1:1

AMPAR, α-amino-3-hydroxy-5-methyl-4-isoxazolepropionic acid receptor; CASPR2, contactin-associated protein-like 2; CSF, cerebrospinal fluid; DPPX, dipeptidyl-peptidase-like protein 6; GABAAR, γ-aminobutyric acid A receptor; GABABR, γ-aminobutyric acid B receptor; GAD65, glutamic acid decarboxylase 65; GlyR, glycine receptor; IgLON5, immunoglobulin-like cell adhesion molecule 5; LGI1, leucine-rich glioma-inactivated 1; mGluR1, metabotropic glutamate receptor 1; mGluR5, metabotropic glutamate receptor 5; NMDAR, N-methyl-D-aspartate receptor.

Due to a lack of therapeutic success, the phenobarbital dosage was reduced to 4.4 mg/kg PO twice daily. In addition, levetiracetam was initiated at 30 mg/kg PO three times daily. At the 4-week follow-up examination, the seizure frequency had decreased from generalized tonic–clonic epileptic seizures occurring approximately every second day to a single generalized tonic–clonic epileptic seizure since initiation of levetiracetam. The previously observed pica had resolved. Residual restlessness and occasional compulsive wandering were reported. On follow-up examination, the cat showed normal gait without evidence of ataxia and unimpaired consciousness. The resolution of the ataxia may have been attributable to the reduction in phenobarbital dosage rather than reflecting improvement of the underlying disease. Further neurological assessment was limited due to limited patient compliance. Follow-up bloodwork revealed mild leukocytosis (11.64 × 10^3^/μL; reference range 6–11 × 10^3^/μL), mildly increased creatine kinase activity (152 U/L; reference range 0–130 U/L), and hyperglycemia (165 mg/dL; reference range 60–100 mg/dL). The phenobarbital serum concentration was 10.9 mg/L (desired therapeutic concentration: 15–35 mg/L). Repeat serum neural antibody testing showed a borderline positive mGluR1 antibody titer of 1:20 (reference < 1:20). A general clinical improvement was noted, and the caretaker reported satisfaction with the clinical status. Immunosuppressive therapy was not initiated because the caretaker declined treatment due to the marked clinical improvement and potential adverse effects. No further follow-up was available because the cat was subsequently lost to follow-up.

## Discussion

In the present case, serum mGluR1 antibodies were identified in a cat with neurological disease characterized pre-dominantly by generalized tonic-clonic epileptic seizures and episodic behavioral abnormalities, including pica, which improved over time, accompanied by a reduction in serum mGluR1 antibody titer. Apart from the elevated serum mGluR1 antibody titer, all other tested neuronal autoantibodies were negative (Table 1), including LGI1 and CASPR2. In humans, the diagnosis of anti-mGluR1 encephalitis is based on a compatible clinical presentation, most commonly subacute cerebellar ataxia, together with detection of mGluR1 antibodies in serum and/or CSF and exclusion of alternative diagnoses. Although mGluR1 antibodies are most commonly detected in both serum and CSF, isolated serum positivity has also been reported (19). Likewise, CSF findings are variable, with mild pleocytosis present in approximately half of patients, whereas increased protein concentrations are less common (19). In the present case, neither mGluR1 antibodies nor pleocytosis nor increased protein concentration were detected in the CSF. However, these findings do not exclude mGluR1-associated autoimmune neurological disease in a cat with neurological signs, as an initially high serum mGluR1 antibody titer was identified, together with the exclusion of other differential diagnoses for epilepsy.

Although anti-mGluR1 encephalitis in humans most commonly presents with a cerebellar syndrome, the clinical presentation in the present cat differed from this pre-dominant phenotype (20). In a recent comprehensive review including 44 human patients with anti-mGluR1 encephalitis, cerebellar ataxia was present in 93.2% of cases, whereas epileptic seizures occurred in only 6.8% and behavioral abnormalities in 20.5%. Reported behavioral abnormalities comprised apathy, paranoia, catatonia, irritability, and mood changes (19).

In contrast to the present case, most patients in the reported human cohort exhibited cerebellar signs in addition to other neurological manifestations. However, a pre-dominantly non-cerebellar presentation has also been described by Vinke et al., (21) who reported a human patient with mGluR1-associated autoimmune encephalitis presenting mainly with epileptic seizures in the absence of cerebellar signs, supporting the concept that anti-mGluR1 encephalitis may extend beyond a purely cerebellar syndrome. This broader clinical spectrum is consistent with the distribution of mGluR1 beyond the cerebellum, particularly within the hippocampal formation, where limbic manifestations including psychiatric symptoms, memory impairment, and seizures may occur (19).

Experimental studies have demonstrated that group I metabotropic glutamate receptors modulate hippocampal excitability and may contribute to epileptogenesis through effects on excitatory synaptic transmission and neuronal network synchronization. Group I metabotropic glutamate receptors in limbic regions such as the hippocampus and nucleus accumbens may contribute to seizure generation and propagation, supporting a potential role of mGluR signaling in limbic dysfunction (22).

The behavioral abnormalities observed in the present case, including pica and compulsive wandering, may further support involvement of limbic or forebrain networks; however, these findings are non-specific and cannot be definitively attributed to mGluR1-associated disease.

In contrast to the hippocampal abnormalities frequently reported in classical feline limbic encephalitis, MRI findings in the present case were unremarkable. This observation is consistent with human anti-mGluR1 encephalitis, in which brain MRI is normal in approximately two-thirds of patients (63.6%), while cerebellar atrophy may become apparent only later during disease progression, indicating that initially normal MRI findings do not exclude the diagnosis (6, 19).

An unusual feature of the present case was the very young age at onset, with seizure episodes reported from approximately 6 weeks of age. Although mGluR1-associated autoimmune encephalitis is pre-dominantly described in adult humans, pediatric cases have also been reported, suggesting that antibody-associated neurological disease may occur across a broader age spectrum (23).

Another notable observation in the present case was the difficulty in achieving therapeutic phenobarbital serum concentrations despite comparatively high dosages and regular administration. Routine diagnostic investigations did not identify clinically relevant comorbidities that could explain altered drug disposition (24, 25). To the authors' knowledge, altered phenobarbital pharmacokinetics have not previously been described in association with mGluR1 antibody-associated disease in humans. Therefore, the unexpectedly low serum concentration observed in the present case remains unexplained.

The present report highlights a possible association between autoimmune mechanisms and epileptic disorders in a cat, even in the absence of obvious structural brain abnormalities on routine diagnostic testing. As this observation is currently based on an isolated case, its clinical relevance remains uncertain. However, increasing awareness of neuronal antibody-associated diseases and broader implementation of antibody testing in veterinary medicine may facilitate identification of additional cases and, over time, help to better define the clinical phenotype, prevalence, and therapeutic relevance of mGluR1-associated disease in cats. Clinical outcomes of human anti-mGluR1 encephalitis are variable; in the largest published cohort, approximately 80% of patients experienced clinical improvement, whereas complete recovery was achieved in only about one-fifth of patients (19).

This case report has several limitations. Phenobarbital peak and trough serum concentrations were not obtained because repeated blood sampling would have required sedation due to the cat's marked defensive behavior. Postprandial bile acid concentration was also not measured. Although fasting bile acid and blood ammonia concentrations were within the normal range, hepatic dysfunction cannot be completely excluded. Furthermore, histopathological confirmation of the underlying disease was not available, which limits definitive conclusions regarding the underlying pathophysiological process. Moreover, repeat MRI examination was not performed. Therefore, despite the initially unremarkable MRI findings, an underlying neurodegenerative disorder cannot be completely excluded, particularly given the young age of the cat. Finally, mGluR1 antibodies were not detected in CSF, which means that an incidental seropositive finding cannot be completely excluded.

A further limitation is that immunosuppressive therapy was not initiated. In human medicine, first-line treatment of anti-mGluR1 encephalitis generally consists of immunotherapy, including high-dose intravenous methylprednisolone, intravenous immunoglobulins, and plasma exchange (19). In the present case, however, immunosuppressive therapy was declined by the caretaker because of the marked clinical improvement observed following optimization of antiseizure therapy and concerns regarding potential adverse effects. Assessment of a potential treatment response was not possible, limiting conclusions regarding a direct pathogenic role of the detected antibodies. In addition, the follow-up period was limited to 4 weeks. Therefore, it remains unclear whether the observed clinical improvement represents sustained remission, a temporary improvement within a fluctuating disease course, or was primarily related to optimization of antiseizure therapy.

## Conclusion

In summary, this case expands the possible clinical spectrum of mGluR1-associated disease in veterinary medicine. Testing for mGluR1 antibodies could be considered as an additional diagnostic tool in cats presenting with epileptic seizures unresponsive to antiseizure medication and concurrent behavioral abnormalities, particularly when routine diagnostic investigations remain inconclusive. However, further studies are required to determine the prevalence, clinical significance, and pathogenic role of mGluR1 antibodies in feline neurological disease.

## Data Availability

The original contributions presented in the study are included in the article/supplementary material, further inquiries can be directed to the corresponding author.
